# Age‐linked biological context shapes adaptive immunity in Alzheimer's disease

**DOI:** 10.1002/alz.71978

**Published:** 2026-11-06

**Authors:** Sophie Shirenova, Arya Biragyn, Eitan Okun

**Affiliations:** ^1^ The Mina and Everard Goodman Faculty of Life Sciences Bar‐Ilan University Ramat Gan Israel; ^2^ The Leslie and Susan Gonda Multidisciplinary Brain Research Center Bar‐Ilan University Ramat Gan Israel; ^3^ The Paul Feder Laboratory for Alzheimer's Disease Research Bar‐Ilan University Ramat Gan Israel; ^4^ Laboratory of Molecular Biology and Immunology, National Institute on Aging Immunoregulation Section National Institutes of Health Baltimore Maryland USA

**Keywords:** adaptive immunity, aging, Alzheimer's disease, B cells, neuroinflammation, T cells

## Abstract

Alzheimer's disease (AD) is increasingly recognized as a neuroimmune disorder with protective or pathogenic adaptive immunity dependent on the biological context. This review synthesizes evidence from human studies, genetic analyses, mouse models, and clinical trials to examine how lymphocytes and humoral immunity influence AD. Human studies show T‐cell accumulation in disease‐relevant brain compartments, peripheral B‐cell remodeling, endogenous anti‐amyloid beta antibodies, and cytokine dysregulation. Complementary genetic studies implicate antigen presentation, lymphocyte regulation, immunoglobulin signaling, and immune checkpoints in AD susceptibility. The findings indicate that the consequences of adaptive immune engagement vary with age and disease stage and are further modified by sex, genetic context, and therapeutic modality. Adaptive immunity in AD is therefore dynamic rather than uniformly beneficial or detrimental. Age in particular emerges as a major determinant that biases immune responses from earlier regulatory states toward later maladaptive inflammation, providing a framework to reconcile conflicting findings and guide stage‐aware immunomodulation.

## Highlights

- Adaptive immunity in AD is dynamic rather than uniformly protective or pathogenic.
- Age and disease stage provide a unifying framework for interpreting adaptive immune responses.
- Genetic, human, and experimental evidence converges on adaptive immunity as a driver of AD.
- Future immunotherapies should be stage‐specific, cell‐targeted, and biologically stratified.

## 1 INTRODUCTION

Alzheimer's disease (AD) remains the leading cause of dementia in older adults and one of the greatest global public health challenges of the 21st century. As of 2025, an estimated 59 million individuals worldwide are living with dementia, of whom ∼60% to 70% are diagnosed with AD. The global prevalence of dementia is expected to rise in parallel with population aging and increased life expectancy.^1^ Clinically, AD is defined by progressive cognitive decline, memory impairment, and loss of functional independence. Pathologically, AD is characterized by systemic^2^ and central^3^ inflammation, synaptic dysfunction,^4^ oligomerization of monomeric peptides, plaques, and fibrils,^5^ extracellular deposition of amyloid beta (Aβ), and intracellular accumulation of hyperphosphorylated tau into neurofibrillary tangles, leading to neuronal loss.^6^ Despite these established hallmarks, the mechanisms driving Aβ and tau accumulation and the resulting neurodegeneration remain unclear. Clarifying how immune processes intersect with these core pathological events is therefore essential to understanding AD progression.

While innate immunity, particularly microglia (the resident myeloid cells of the central nervous system [CNS]), has attracted the most attention, recent findings emphasize the importance of adaptive immunity in both promoting and protecting against AD. Brain‐derived antigens drain via the glymphatic‐meningeal lymphatic systems into cervical lymph nodes (cLNs), where they are presented to adaptive immune cells, such as T and B cells.^7^, ^8^ Activated adaptive immune cells infiltrate sites of inflammation in the brain and modulate the activity of microglia, oligodendrocytes, and neurons. Complementing these mechanistic observations, genome‐wide association studies (GWASs) implicate adaptive immunity in AD by identifying risk loci within genes that regulate immune functions, including antigen presentation, T‐cell function, and immunoglobulin (Ig) activity.^9^, ^10^ Together, these findings support adaptive immunity as an active component of AD pathogenesis, yet its precise role in disease initiation and progression remains unresolved.

Adaptive immunity is mediated primarily by antigen‐specific T and B lymphocytes, which generate targeted and memory responses. Immune‐based intervention strategies have become a major focus of therapeutic development and experimental investigation in AD. Clinical trials designed to modulate these pathways have produced conflicting outcomes, with some studies reporting clinical or biomarker improvement^11^, ^12^ and others demonstrating limited efficacy or treatment‐related adverse effects.^13^, ^14^ Although these studies show that immune intervention is feasible in AD, conflicting outcomes may reflect pathogenic adaptive immune activation shaped by treatment modality, disease stage, biological age, and genetic risk. These divergent outcomes are mirrored in preclinical AD studies, where manipulation of adaptive immune components in transgenic mouse models, through genetic deficiencies or antibody‐mediated depletion, attenuates amyloid pathology at later stages of the disease,^15^, ^16^, ^17^ whereas similar interventions exacerbate amyloid deposition or accelerate disease progression when applied at early stages or in younger animals.^18^, ^19^, ^20^, ^21^ Together with the heterogeneous outcomes observed in clinical trials, these findings indicate that the effects of adaptive immune modulation in AD are highly context‐dependent. This raises a central unresolved question: Under what pathophysiological conditions does adaptive immunity confer protective rather than pathogenic effects in AD?

Emerging evidence indicates that biological age is a critical determinant of whether adaptive immune responses mitigate or amplify neurodegeneration across disease stages.^15^, ^21^, ^22^, ^23^ Adaptive immune cells change both phenotypically and functionally during aging, accumulating T and B cells exhibiting chronically activated, exhausted, and/or immunosenescent features that are not present or rarely found in young hosts.^24^, ^25^, ^26^ However, the cellular and temporal dynamics of B‐ and T‐cell activity throughout AD progression remain poorly understood, limiting our ability to define how disease stage and individual patient factors influence these outcomes. These observations support the hypothesis that the role of adaptive immunity in AD is not fixed but varies with age, disease stage, and genetic background.

Untangling context‐dependent immune effects in patients remains challenging because most human studies capture disease near symptom onset and are confounded by heterogeneity in genetic background, immune status, and comorbidities. To overcome these limitations, preclinical early‐onset AD (EOAD) models carrying *amyloid precursor protein* (*APP*), *presenilin‐1* (*PS1*), or *presenilin‐2* (*PS2*) mutations provide a controlled framework for examining immune‐brain interactions across defined pathological stages. These models reveal strain‐, sex‐, and age‐specific differences in adaptive immune cell infiltration, Ig deposition, and microglial interactions,^20^, ^21^, ^22^, ^23^ offering mechanistic insight into the conditions under which adaptive immunity becomes protective or pathogenic.

In this review, we integrate evidence from human studies, genetic analyses, mechanistic mouse models, and clinical immunotherapy trials to examine how adaptive immunity shapes AD development and progression. We argue that age and disease stage provide the most consistent framework for reconciling apparently contradictory findings, while sex, genetic context, and syndrome‐specific states, such as Down syndrome (DS), further modify adaptive immune behavior. EOAD‐like mouse models are discussed not as the sole disease frame but as mechanistic systems that reveal when adaptive immunity supports surveillance and clearance and when it shifts toward maladaptive inflammation.

## 2 HUMAN EVIDENCE AND CONCEPTUAL BASIS FOR ADAPTIVE IMMUNITY IN AD

Human studies provide evidence that adaptive immunity is altered throughout AD and offer a conceptual framework for understanding how these changes may contribute to disease pathogenesis.

### 2.1 Age‐driven remodeling of adaptive immunity

The adaptive immune system evolves throughout life, acquiring memory for antigens and responding with increasing affinity and kinetics upon re‐exposure. During aging, adaptive immune cells undergo phenotypic and functional changes that can impair immune competence, which may contribute to AD pathogenesis.^27^ Aging shapes both composition and responses of the innate and the adaptive immune systems, leading to the accumulation of antigen‐experienced B‐ and T‐cell subsets, including age‐associated B cells (ABCs),^24^ pathogenic insulin resistance‐inducing B cells (4BL cells),^25^ and a distinct CD8^+^ T‐cell subset (DP8 cells) co‐expressing the ectonucleotidases CD39 and CD73.^26^ Aging drives a shift in T‐helper (Th) CD4^+^ and cytotoxic CD8^+^ T cells from naïve phenotypes toward effector‐memory states, characterized by heightened activation and increased expression of interferon‐gamma (IFN‐γ), tumor necrosis factor‐alpha (TNF‐α), and cytotoxic molecules. These age‐associated changes are evident across multiple tissues, including the spleen and peripheral blood in both humans and mice.^28^, ^29^, ^30^ Aging is also characterized by a chronic, systemic, low‐grade inflammatory state, termed inflammaging.^31^ Inflammaging affects multiple physiological systems, including the adaptive immune compartment, and creates a peripheral environment that can influence CNS homeostasis.^31^ Importantly, such age‐related remodeling may also extend to antigen‐specific adaptive immune responses during neurodegenerative disease. In Parkinson's disease, α‐synuclein‐specific T‐cell responses have been identified and were subsequently shown to peak during the preclinical and early symptomatic stages before declining with disease progression.^32^, ^33^ Although observed in a distinct neurodegenerative disorder, this temporal pattern provides independent evidence that antigen‐specific adaptive immune responses can evolve across disease stages.

### 2.2 Glymphatic antigen drainage to peripheral immunity

In neurodegenerative proteinopathies, a group of disorders characterized by abnormal protein accumulation or misfolding in tissues (e.g., AD, Parkinson's disease, and frontotemporal dementia), pathological antigens originating in the brain are thought to drain via the glymphatic system.^34^, ^35^ The glymphatic system is a proposed clearance pathway through which interstitial fluid and cerebrospinal fluid (CSF) exchange, facilitating the transfer of brain‐derived proteins to the meningeal lymphatic vessels and eventually to the deep cLNs (dcLNs).^8^, ^36^ In AD, these brain‐derived proteins include toxic Aβ and pathological tau species, which disrupt cellular homeostasis and drive neurodegeneration. In addition to drainage toward the cervical lymph nodes, immune surveillance of CNS‐derived signals also occurs locally at the brain borders. The dural sinuses function as neuroimmune interfaces where CNS‐derived antigens can be sampled by resident immune cells, while skull bone marrow provides a local source of immune cells that can access the meninges through specialized vascular channels.^37^, ^38^ Within the dcLNs, adaptive immune cells interact with antigen‐presenting cells (APCs), including dendritic cells and B cells, which display brain‐derived peptides to naïve CD4^+^ and CD8^+^ T cells, thereby triggering their activation.^39^ Following antigen exposure, B cells, acting as Ig producers, cytokine producers, and APCs, undergo clonal expansion along with activated T cells to promote a coordinated adaptive immune response.^40^ Through this pathway, adaptive immunity can continuously survey CNS‐derived antigens and mount antigen‐specific responses. Evidence from human AD patients indicates that Aβ‐containing cells are present in the dcLNs and are absent in the inguinal LNs.^41^, ^42^

### 2.3 T‐cell infiltration, clonal expansion, and antigen recognition in human AD

T cells are not randomly distributed in the AD brain but are localized to anatomically and pathologically relevant regions. This pattern is supported by the increased number of CD3^+^ T cells seen in the brains of human AD patients, which is significantly higher than in other neurodegenerative disorders.^43^, ^44^ Among these, infiltrating CD8^+^ T cells were observed in the hippocampus, middle temporal gyrus, perivascular spaces, and leptomeninges.^45^, ^46^, ^47^ Adaptive immune cells were also found adjacent to Aβ plaques and phosphorylated tau tangles, in the brain parenchyma of both human patients and mouse models.^15^, ^20^, ^45^, ^48^, ^49^ In a separate analysis, CD8^+^ T cells were identified clustered around hippocampal perivascular spaces affected by cerebral amyloid angiopathy (CAA).^46^ The heightened T‐cell infiltration in CAA‐affected vessels may reflect entry through vascular breaches rather than a response to Aβ itself. Because tau pathology was not assessed in that study, it remains unclear whether T cells in these regions are more strongly associated with Aβ or tau pathology.

Quantitative analyses further refine this association. In human brain tissue, CD3^+^ T cells were significantly more abundant in the frontal gyrus of individuals with Braak stage VI, characterized by extensive neocortical tau pathology, than those with preclinical Braak stages I to II, in which neurofibrillary tangles are confined mainly to the transentorhinal/entorhinal cortex.^50^, ^51^ These findings are consistent with recent evidence indicating that T‐cell infiltration is more pronounced in brains affected by tau pathology, in both human AD and experimental tauopathy models, supporting a particularly strong association between T‐cell accumulation and tau‐associated neurodegeneration.^52^ Importantly, in the PS19 tauopathy model, T‐cell depletion reduced neuroinflammation, brain atrophy, and cognitive impairment, providing direct evidence that infiltrating T cells can contribute causally to tau‐associated neurodegeneration.^51^ Similarly, a positive correlation was found between CD3^+^ T‐cell numbers and tau burden in non‐immunized AD patients, independently of Aβ_42_, microglial markers, or complement C1q.^53^ These data collectively suggest a tighter relationship between T‐cell accumulation and tau pathology than with amyloid deposition.

A clonal T‐cell response is also evident, characterized by the expansion of T‐effector memory CD45RA^+^ CD8^+^ T cells (termed TEMRA).^47^ These highly differentiated cytotoxic T cells expand during aging and chronic inflammation and are enriched in AD. As late‐stage senescent cells, TEMRA cells can exhibit heightened cytotoxicity and increased IFN‐γ production.^46^, ^54^ Notably, the antigen specificity of these T‐cell responses remains incompletely defined, although Aβ‐reactive T cells have been detected in humans and were more frequent in older individuals and patients with AD than in middle‐aged controls.^55^ Evidence from in vitro studies further refines this question, showing that CD4**^+^** and CD8**^+^** T cells respond to the C‐terminus and mid regions, but not to the N‐terminus of the Aβ or tau peptides.^54^ The reason for this selective epitope responsiveness remains unknown. However, peptide reactivity alone does not demonstrate that these epitopes drive the dominant clonal expansions observed in vivo. Consistent with this, T‐cell receptor (TCR) analysis in AD patients failed to identify Aβ‐specific clones and instead detected Epstein‐Barr virus‐specific CD8^+^ TCRs.^46^ These findings suggest that antigen specificity in AD may extend beyond classical amyloid‐directed recognition and could reflect a more complex interaction with pathogen‐derived antigens.^46^ Thus, although T cells accumulate and clonally expand in AD, the precise antigenic drivers of this response remain unresolved.

### 2.4 Peripheral B‐cell remodeling and antibody responses in human AD

Compared with T cells, B cells are rarely detected in the brain of AD patients and are typically localized to the meninges, perivascular spaces, draining dcLNs, and circulation rather than the brain parenchyma.^48^ Despite this limited parenchymal presence, evidence indicates that B cells have an active role in AD pathology. Whereas healthy aging is characterized by a decline in peripheral B‐cell numbers, patients with AD do not exhibit this reduction.^49^ Moreover, increased Aβ plaque load was associated with elevated peripheral B‐cell counts, but not with T‐ or natural killer (NK)‐cell counts. These observations suggest that B‐cell alterations in AD reflect disease burden rather than normal aging.

Transcriptomic analyses revealed altered expression of B‐cell‐specific pathways in AD, including genes linked to lineage commitment, isotype switching, and circulating Ig complexes.^49^ These analyses further reveal upregulation of interleukin (IL)‐10 and recombination activating gene 2 (RAG2), indicating chronic activation and ongoing receptor recombination, alongside reduced expression of forkhead box P3 (FOXP3) and replication timing regulatory factor 1 (RIF1), suggesting impaired regulatory signaling. Differential expression of polymeric Ig receptor (PIGR) further reflects altered antibody transport pathways,^49^ as PIGR mediates the transcytosis of polymeric Igs (e.g., IgA and IgM) across epithelial and barrier tissues, implying that its dysregulation may disrupt Ig trafficking between peripheral compartments and barrier interfaces, including those relevant to CNS immune surveillance.

These observations suggest that in AD, B cells are numerically altered and undergo transcriptional reprogramming that changes their cytokine output, receptor recombination, regulatory potential, and antibody transport. Early in the disease, this may enhance beneficial antibody‐mediated microglial clearance of Aβ. However, chronic dysregulation, characterized by excess IL‐10, reduced regulatory FOXP3*^+^* cells, possible autoreactivity driven by RAG2 activity, and impaired antibody transport due to altered PIGR expression, could shift B cells toward pathogenic roles, amplifying dysfunction rather than promoting homeostasis. This proposed temporal shift reflects the broader transition from protective surveillance to maladaptive amplification proposed for adaptive immunity in AD.

Consistent with this functional duality, B‐cell‐derived Igs have been shown to modulate microglial activity by enhancing Aβ phagocytosis during acute exposure but promoting microglial dysfunction and increased Aβ accumulation under chronic stimulation.^49^ As a result, AD patients spontaneously develop anti‐Aβ antibodies or exhibit increased numbers of B cells producing anti‐Aβ antibodies.^56^ Thus, antibody production in AD appears to reflect both adaptive engagement and dysregulation that may occur with chronic exposure.

This pattern may also be influenced by age‐related changes in the B‐cell compartment. ABCs are antigen‐experienced autoreactive B cells characterized as B220^+^CD19^+^Tbet^+^CD11c^+^CD21^−^CD23^−^CD95^−^CD43^−^ and accumulate with aging in various tissues, including the meninges.^24^, ^57^ ABCs require constitutive B‐cell receptor (BCR) activation, such as chronic exposure to self‐antigens in autoimmune conditions. This raises the hypothesis that the B‐cell response in AD could reflect ABCs reacting to Aβ, following constant Aβ drainage to the meninges and dcLNs, thereby integrating an additional aging‐dependent mechanism into AD pathogenesis.^58^ If so, B‐cell activation in AD may represent an intersection between chronic antigen exposure and age‐dependent immune remodeling.

### 2.5 Microglia‐adaptive immunity crosstalk

Within the brain, microglial cells serve as myeloid sentinels that continuously survey their surroundings and rapidly shift their transcriptional programs in response to sensed perturbations.^59^ A central regulator of these microglial responses is triggering receptor expressed on myeloid cells 2 (TREM2), an AD risk gene that regulates microglial survival, metabolism, phagocytosis, and the transition toward disease‐associated activation states.^60^ Through these effects, TREM2 can shape the inflammatory and antigen‐presenting environment encountered by infiltrating lymphocytes, thereby providing an important mechanistic link between AD‐associated microglial states and adaptive immune responses. Because Aβ is neurotoxic, microglia initially respond by attempting to clear it through phagocytosis and enzymatic degradation.^59^ Under acute activation, this response is adaptive and effective, reducing Aβ burden. However, chronic or sustained stimulation drives microglia into a disease‐associated state characterized by maladaptive pro‐inflammatory signaling.^61^ In this state, microglia exhibit diminished capacity to clear Aβ and instead amplify local inflammatory cascades, exacerbating neuronal stress and tissue injury.^59^, ^61^

Another source of microglial activation is the secretion of pro‐inflammatory cytokines, such as IFN‐γ, by infiltrating T cells,^62^ which in the aged brain is predominantly produced by CD8^+^ T cells and, to a lesser extent, by effector CD4^+^ T cells, thereby altering the microglial phenotypic state and function,^54^, ^63^, ^64^ although the direct mechanistic interplay between IFN‐γ‐producing T cells and microglia in the context of AD remains to be fully defined.^65^ Beyond IFN‐γ, IL‐17‐producing γδ T cells may represent an additional T‐cell‐mediated pathway contributing to early dysfunction in AD. In female 3xTg‐AD mice, IL‐17‐producing cells, predominantly γδ T cells, accumulated in the brain and meninges concomitantly with the onset of cognitive decline, while intracerebroventricular IL‐17 neutralization prevented early deficits in short‐term memory and synaptic plasticity.^66^ Notably, these effects preceded blood–brain barrier (BBB) disruption and detectable Aβ or tau pathology, suggesting that meningeal γδ T‐cell‐associated IL‐17 signaling can contribute to early functional impairment before established protein deposition. Together, inflammaging and age‐altered adaptive immune activity create a pro‐inflammatory landscape that shapes microglial responses and contributes to the escalating neuroimmune dysfunction characteristic of AD.^31^, ^59^, ^61^, ^65^

Whereas studies utilizing mouse models of amyloidosis report Aβ‐reactive CD8^+^ T cells infiltrating the brain following immunization,^17^ it remains unclear whether Aβ, tau pathology, or broader inflammatory cues drive the recruitment of these T cells to the brain in AD patients. Resolving these questions will be essential to defining how adaptive immune signals influence microglial dysfunction and disease progression in AD.

## 3 GENETIC EVIDENCE SUPPORTING ADAPTIVE IMMUNE INVOLVEMENT IN AD

While most human genetic evidence derives from late‐onset AD (LOAD) cohorts, these studies implicate antigen presentation, lymphocyte regulation, and Ig‐related pathways in AD susceptibility. The strongest support for adaptive immune involvement comes from genome‐wide association studies of LOAD.

### 3.1 GWAS‐based evidence for adaptive immune pathway involvement in AD

Although EOAD is primarily linked to rare mutations in *APP*, *presenilin (PSEN)1*, or *PSEN2*, GWASs of LOAD have identified more than 130 risk loci,^9^ with key enriched loci in innate and adaptive immune pathways^9^, ^10^, ^67^ (Table 1, Figure 1).

**TABLE 1:** Selected AD‐associated loci linking adaptive immune and immune‐regulatory pathways to disease susceptibility.

Gene / locus	Encoded protein	Immune function	Implication in AD	Odds ratio	Reference
*APOE* rs429358‐C (ε4‐defining allele)	Apolipoprotein E	Lipid transport, Aβ clearance, synaptic and immune regulation	Strongest genetic risk factor for LOAD; broad impact on immune and metabolic pathways	3.32 (C vs T allele)	^68^
PLCG2 rs72824905‐G (P522R)	Phospholipase Cγ2	Immune‐receptor signaling, including BCR‐ and Fc‐receptor‐associated pathways	Rare coding variant associated with reduced AD risk; links immune‐receptor signaling to AD susceptibility	0.74	^67^
ABI3 rs616338‐T (S209F)	ABI family member 3	Actin‐cytoskeletal and immune‐cell signaling	Rare coding variant associated with increased AD risk; implicates immune‐cell regulatory pathways in disease susceptibility	1.32	^67^
HLA‐DRB1*04:04/*04:01	MHC class II DR β‐chain	Antigen presentation to CD4^+^ T cells	Protective HLA alleles associated with reduced AD risk and tau‐related phenotypes, supporting a role for antigen presentation	0.86 / 0.93	^69^
*HLA‐DQA1 rs6605556‐G*	MHC class II DQ α‐chain	Antigen presentation to CD4^+^ T cells	Protective HLA‐region association reinforces the contribution of MHC‐II‐mediated adaptive immune pathways	0.91	^67^
SPI1 rs10437655‐A	PU.1 transcription factor	Regulates differentiation and transcriptional programs in myeloid and lymphoid cells	AD‐associated locus linking immune‐cell transcriptional regulation to disease susceptibility	1.06	^67^
LILRB2 rs587709‐C	Leukocyte immunoglobulin‐like receptor B2	Inhibitory ITIM‐dependent immune signaling and regulation of activation thresholds	Risk‐associated variant implicating inhibitory immune‐receptor signaling in AD	1.05	^67^
TNIP1 rs871269‐T	TNF‐α‐induced protein 3‐interacting protein	Negative regulation of NF‐κB‐dependent inflammatory signaling	Protective AD association linking regulation of inflammatory signaling to disease risk	0.96	^67^
CR1 rs679515‐T	Complement receptor 1	Complement‐mediated antigen handling; expressed by immune populations including B cells	Strong AD association connecting complement‐mediated immune regulation with disease susceptibility	1.13	^67^
INPP5D rs10933431‐G	SHIP1	Inhibitory PI3K signaling downstream of immune receptors	Protective association linking immune‐receptor signaling thresholds to AD susceptibility	0.93	^67^
CLU rs11787077‐T	Clusterin	Extracellular chaperone, complement regulation, and immune‐mediated antigen handling	Protective association linking complement/immune regulation with AD susceptibility	0.91	^67^

Abbreviations: ABI3, ABI family member 3; AD, Alzheimer's disease; APOE, apolipoprotein E; BCR, B‐cell receptor; CLU, clusterin; CR1, complement receptor 1; HLA, human leukocyte antigen; HLA‐DQA1, HLA class II histocompatibility antigen DQ α1 chain; HLA‐DRB1, HLA class II histocompatibility antigen DR β1 chain; INPP5D, inositol polyphosphate‐5‐phosphatase D; ITIM, immunoreceptor tyrosine‐based inhibitory motif; LILRB2, leukocyte immunoglobulin‐like receptor B2; LOAD, late‐onset Alzheimer's disease; MHC, major histocompatibility complex; NF‐κB, nuclear factor kappa B; PLCG2, phospholipase C gamma 2; SHIP1, Src homology 2 (SH2) domain‐containing inositol 5′‐phosphatase 1; SPI1/PU.1, Spi‐1 proto‐oncogene/PU.1 transcription factor; TNIP1, TNFAIP3‐interacting protein 1.

**FIGURE 1:**
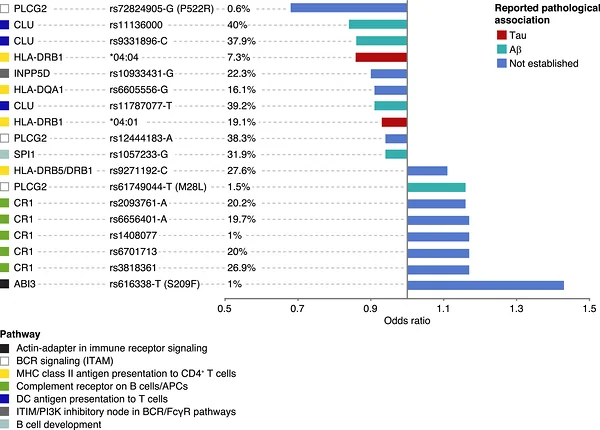
Genetic variants linking adaptive immune pathways to AD risk. Summary of selected AD‐associated genetic variants mapped to adaptive immune and immune regulatory pathways. Bars show odds ratios for each variant, with values below 1 indicating reduced risk and values above 1 indicating increased risk. Percentages indicate the frequency of the effect allele. Bar color denotes reported pathological association: tau, Aβ, or not established. The pathway annotation highlights convergence on BCR (B‐cell receptor) signaling, which is mediated by immunoreceptor tyrosine‐based activation motifs (ITAM), MHC class II antigen presentation, complement receptor activity, dendritic cell antigen presentation, immunoreceptor tyrosine‐based inhibitory motif (ITIM)‐ and PI3K‐mediated inhibitory signaling, and B‐cell development.

The strongest genetic risk factor for LOAD remains the apolipoprotein E (*APOE)* ε4 allele, which affects Aβ clearance, lipid metabolism, synaptic signaling, mitochondrial homeostasis, and immune responses.^9^, ^10^, ^70^ With respect to immune function, *APOE* ε4 has been linked to microglial activation,^71^ immunosuppression of IL‐17^+^ neutrophils,^72^ lipid‐metabolism disturbances, and BBB dysfunction, which together promote T‐cell infiltration to the brain.^73^

Beyond *APOE*, GWASs have identified several loci that further strengthen the link between adaptive immunity and AD.^10^ One of the most prominent is *human leukocyte antigen class II DR beta 1 (HLA‐DRB1)*, which encodes a major histocompatibility complex class II (MHC‐II) molecule expressed on APCs. Genetic associations involving both risk‐associated and protective HLA‐DRB1 alleles further implicate MHC‐II‐mediated antigen presentation in AD susceptibility and disease modification, potentially by reshaping CD4^+^ T‐cell activation against CNS‐derived antigens.^9^, ^52^

Another key locus is the *spi‐1 proto‐oncogene* (*SPI1*, also termed PU.1), a transcription factor involved in myeloid and lymphoid development, including APCs.^74^ *SPI1* regulates immune genes involved in antigen presentation and T‐cell priming. Reduced expression of *SPI1* has been linked to delayed AD onset and lower disease risk,^9^, ^74^ potentially through reduced microglial activation and inflammatory signaling.

Additional AD‐associated loci summarized in Table 1 and Figure 1, including *PLCG2*, *INPP5D*, *HLA‐DQA1*, *LILRB2*, *TNIP1*, *CR1*, *CLU*, and *ABI3*, converge on immune‐receptor signaling, complement regulation, antigen handling, and immune‐cell activation.^67^, ^68^, ^69^, ^75^, ^76^, ^77^, ^78^, ^79^ Although several of these genes have prominent functions in myeloid cells, the pathways they regulate also shape the broader immune environment in which adaptive immune responses develop.

### 3.2 Immune regulatory mechanisms associated with AD risk

Leukocyte Ig‐like receptor genes, including *LILRB2* and *LILRB4*, are associated with LOAD and regulate activation thresholds in myeloid and lymphoid cells.^10^, ^80^, ^81^ Notably, *LILRB2* has been shown to interact with Aβ in mouse models, and antibody‐mediated inhibition reduces amyloid burden, linking immune inhibitory signaling to amyloid pathology.^82^ Another GWAS locus, TNF‐α‐induced protein 3 interacting protein (*TNIP1*), encodes a negative regulator of nuclear factor kappa B signaling.^83^ Through this pathway, TNIP1 may shape the inflammatory environment that influences antigen presentation and adaptive immune responses.^84^

Genetic evidence also implicates immune checkpoint regulation in AD susceptibility. Hepatitis A virus cellular receptor 2 (*HAVCR2*), also known as T‐cell Ig and mucin‐domain containing‐3 (TIM‐3), encodes an inhibitory receptor expressed on both T cells and microglia.^85^ TIM‐3 regulates T‐cell exhaustion and peripheral tolerance and in the CNS also modulates microglial activation. In microglia, TIM‐3 signaling suppresses pro‐inflammatory responses while concurrently limiting phagocytic and antigen‐presenting capacity. Conversely, altered TIM‐3 expression in LOAD brains suggests dysregulated checkpoint control, which may either reinforce maladaptive immune suppression or impair the resolution of chronic neuroinflammation.^10^, ^85^

Complement signaling further integrates innate and adaptive immune pathways in AD. Specifically, complement receptor 1 (*CR1*) binds complement‐opsonized targets such as C3b‐ or C4b‐tagged Aβ aggregates, thereby promoting their recognition and clearance by phagocytic cells, including microglia and peripheral myeloid cells. *CR1*‐mediated uptake of complement‐coated antigens may also facilitate MHC‐II loading and CD4^+^ T‐cell priming, linking amyloid clearance to adaptive immune activation. Thus, *CR1* risk variants associated with AD may disrupt the balance between efficient opsonization‐driven clearance and controlled immune activation.^9^, ^86^

This genetic evidence points to an integrated immune circuit in AD, linking complement activation, antigen handling, MHC‐II‐mediated presentation, T‐cell priming, checkpoint regulation, and microglial inflammatory state (Table 1). Rather than implicating isolated immune pathways, these findings indicate that genetic variation may shift the balance between protective clearance and chronic neuroinflammation.

## 4 EVIDENCE FROM EOAD MOUSE MODELS FOR ADAPTIVE IMMUNE INVOLVEMENT IN AD

Most causal insights into adaptive immunity in AD have emerged from EOAD‐like transgenic and knock‐in mouse models. Although these models incompletely recapitulate the full human disease spectrum, particularly tau‐dominant and late‐life features, they remain indispensable for defining how lymphocytes, antibodies, and microglia interact across disease progression.

### 4.1 Mutation landscape and timeline of pathophysiological changes in EOAD mouse strains

While recently developed mouse models better recapitulate familial EOAD in humans by incorporating both Aβ and tau pathologies (see comprehensive review^87^), their use in studies of adaptive immunity remains limited. Most investigations of adaptive immune involvement have therefore relied on first‐generation EOAD mouse models, which predominantly feature Aβ pathology with minimal or no tau involvement. This raises a broader consideration regarding how the absence of extensive tau pathology might shape the T‐cell phenotypes observed in these models, particularly when compared with the more complex, stage‐dependent immune signatures observed in human AD. Thus, the central question is whether immune phenotypes defined in Aβ‐dominant systems fully capture the adaptive immune dynamics of human disease.

Despite their limitations, first‐generation models have been instrumental in uncovering fundamental aspects of adaptive and innate immune responses in AD. Although some of the discussed models were extensively reviewed elsewhere,^87^, ^88^, ^89^ here, we briefly outline only the features most relevant to interpreting adaptive immune findings: pathological tempo, promoter context, Aβ/tau involvement, and timing of neuroinflammatory change (Table 2, Figure 2). For an overview of findings in mouse models that develop tau pathologies, see.^90^

**TABLE 2:** Comparative genetic, neuropathological, and behavioral characteristics of widely used EOAD transgenic and knock‐in mouse models.

Mouse strain	5xFAD	APPswe/PS1^ΔE9^	APP/PS1^L166P^	APP^NL‐G‐F^	3xTg‐AD
Genetic background	C57BL/6J	C57BL/6J	C57BL/6J	C57BL/6J	mixed B6;129 background
Mutations/transgenes	hAPP (Swedish K670N/M671L, Florida I716V, London V717I) + PS1 (M146L, L286V)	hAPP695swe (K595N/M596L) + PS1^ΔE9^	hAPP (K670N/M671L) + PS1 (L166P)	hAPP Knock‐in: Swedish (K670N/M671L), Iberian (I716F), Arctic (E693G)	hAPP Swedish (K670N/M671L) + PS1 (M146V) + tau (P301L)
Promoter	Thy1	PrP (neuronal & astrocytic)	Thy1	Endogenous APP	Thy1.2
Earliest Aβ pathology	Intracellular Aβ ↑ at 1.5 months; extracellular plaques in cortex by 2 months; hippocampus by 6 months	Elevated Aβ at 2.5 months; extracellular plaques in cortex/hippocampus from 4 months	Aβ plaques in cortex at 1.5 months, hippocampus by 4 months	Soluble/insoluble Aβ_1__−__42_ ↑ at 1 month; plaques by 4 months	Intraneuronal Aβ in cortex by 3 to 4 months; plaques by 6 months
Gliosis onset	Microgliosis at 4 months; astrogliosis in hippocampus by 12 months, cortex by 18 months	Microgliosis by 6 months; astrogliosis (↑GFAP) by 9 months	Hypertrophic microglia from 1.5 months	Micro‐ and astrogliosis from 9 months	Astrocyte reactivity near plaques by 12 months
Synaptic/LTP deficits	LTP ↓ and synaptophysin ↓ from 4 months	Dendritic loss in amygdala by 4 months; no hippocampal neurodegeneration up to 17 months	LTP is disrupted from 15 months	LTP intact at 3 to 4 months, impaired by 6 to 8 months	Synaptic transmission deficits by 6 months
Neuronal loss	Pyramidal neuron loss in cortex/subiculum by 9 months	None in the hippocampus	Neuronal loss in the dentate gyrus by 17 months	Synaptophysin, PSD95 ↓ near plaques (onset n.s.)	Neuronal loss detectable from 12 months
Behavioral deficits	Memory impairment (fear conditioning, Y‐maze, MWM) from 4 months	MWM deficits by 3.5 months	Spatial memory deficits from 8 months	Behavioral changes from 6 months (↓exploration, learning, memory)	Spatial learning and fear conditioning deficits by 6.5 months
Distinctive features	Rapid, aggressive Aβ pathology; strong neuroinflammation; early immune alterations	Slower progression; late intracellular Aβ (15 months); extensive cortical spread by 10 months	Early cortical Aβ with late functional loss; strong microglial activation	Physiological APP expression (no overexpression artifacts); gradual pathology	Dual Aβ and tau pathology; translational model for combined AD hallmarks

Abbreviations: ↓, decrease; ↑, increase; APP, amyloid precursor protein; GFAP, glial fibrillary acidic protein; hAPP, human APP; LTP, long‐term potentiation; MAPT, microtubule‐associated protein tau; MWM, Morris water maze; n.s., not specified; PSD95, postsynaptic density protein 95; PS1, presenilin‐1; PrP, prion protein promoter; tau/NFT, tau neurofibrillary tangles;.

**FIGURE 2:**
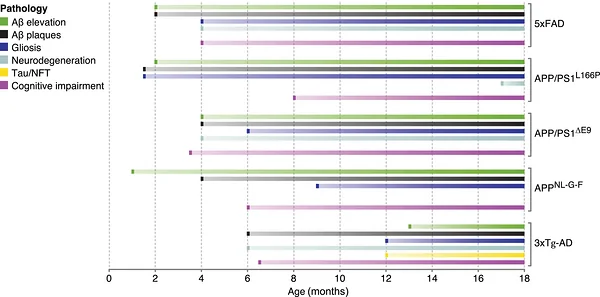
Temporal emergence of pathological features across AD mouse models. Comparative timeline showing the reported onset of major AD‐related pathological and functional features across 5xFAD, APP/PS1^L166P^, APP/PS1^∆E9^, APP^NL‐G‐F^, and 3xTg‐AD mouse models. The scheme summarizes the age at which Aβ elevation, Aβ plaque deposition, gliosis, neurodegeneration, cognitive impairment, and tau or neurofibrillary tangle pathology are detected. Marked model‐specific differences are evident in disease kinetics, with some strains showing early amyloid and inflammatory changes, while others display later neurodegenerative or tau‐associated pathology. Colored bands begin at earliest reported onset and darken to the right to indicate progressive intensification with age.

#### 4.1.1 5xFAD: Rapid Aβ accumulation and early neuroimmune activation

The five familial AD mutations (5xFAD) mice develop rapid and aggressive Aβ pathology, with early plaque formation followed by gliosis and behavioral impairment^45^, ^91^ (Table 2, Figure 2). This compressed disease course makes the model useful for studying early neuroimmune activation and adaptive immune recruitment during Aβ‐driven pathology. However, this model predominantly reflects Aβ‐driven pathology with minimal tau involvement, which may limit its ability to capture the more complex, stage‐dependent immune dynamics observed in human AD.

#### 4.1.2 APP/PS1^L166P^: early plaque deposition with delayed functional decline

APP/PS1^L166P^ mice^92^ exhibit early cortical Aβ deposition and microglial activation, making them useful for examining immune responses during the initial stages of plaque‐associated pathology (Table 2, Figure 2). Yet, functional decline occurs later, which may complicate temporal alignment between amyloid pathology, immune activation, and neurodegeneration.^93^, ^94^

#### 4.1.3 APP/PS1^ΔE9^: gradual Aβ accumulation with region‐specific neurodegeneration

APPswe/PS1^ΔE9^ mice^95^ show a slower progression of Aβ deposition and gliosis than 5xFAD mice, allowing immune responses to be examined across a more gradual pathological trajectory^96^, ^97^, ^98^, ^99^, ^100^ (Table 2, Figure 2). Nevertheless, this model shows region‐specific and incomplete neurodegeneration, which may limit its ability to fully capture neurodegenerative progression in human AD.^101^, ^102^, ^103^

#### 4.1.4 APP^NL‐G‐F^: physiological Aβ expression with gradual functional impairment

APP^NL‐G‐F^ mice^104^ avoid artifacts related to hAPP overexpression by introducing AD‐linked mutations into the endogenous APP locus. This model develops gradual Aβ pathology and later neuroinflammatory changes, providing a useful system for studying immune responses under more physiological APP expression^105^, ^106^ (Table 2, Figure 2). However, the timing of neurodegeneration is not clearly defined.^104^, ^105^, ^106^, ^107^

#### 4.1.5 3xTg‐AD: sequential Aβ and tau pathology

3xTg‐AD mice^108^, ^109^, ^110^ incorporate both Aβ‐ and tau‐related pathology, making them valuable for examining adaptive immune responses in a model that includes both major AD hallmarks (Table 2, Figure 2). Yet the delayed onset of tau pathology relative to early Aβ accumulation may complicate the interpretation of stage‐specific immune responses across disease progression.^109^, ^111^, ^112^, ^113^

In addition to the mutation profile, promoter context, such as *Thy1*, *PrP*, or endogenous knock‐in expression, critically influences the spatial distribution, expression level, and temporal kinetics of the pathology. Although these strains broadly converge on increased Aβ production, they differ substantially in the timing and severity of downstream pathological features, including intracellular and extracellular Aβ accumulation, plaque formation, microgliosis and astrogliosis, synaptic and long‐term potentiation deficits, neuronal loss, behavioral impairment, and immune landscape. This variability reflects multiple factors, including differences in genetic background, transgene expression levels, promoter specificity, methodological approaches, and, critically, the age at intervention and assessment. Accordingly, conclusions regarding AD mechanisms and immune dynamics require careful cross‐model interpretation rather than direct extrapolation.

### 4.2 Characteristics of adaptive immune response in EOAD mouse models

Understanding the adaptive immune landscape in EOAD mouse models requires dissecting the complex interactions among immune cells at the interface between CNS and peripheral tissues. Although growing evidence implicates adaptive immunity in AD progression, systematic, age‐resolved characterization of adaptive immune responses across EOAD mouse models remains limited, which constrains cross‐model comparisons. This gap complicates the interpretation of model‐specific immune signatures and their translational relevance to human AD. In this section, we synthesize the current state of the art to provide an updated framework for identifying strain‐, mutation‐, and age‐dependent factors that may shape adaptive immune responses and influence the outcome of immune‐targeted interventions.

Among first‐generation models, 5xFAD mice exhibit pronounced yet partially conflicting alterations in adaptive immunity, particularly in the meninges and brain parenchyma. For example, aged male 5xFAD mice (13 to 14 months old) exhibit reduced lymphatic coverage along the dural sinuses, accompanied by increased numbers of T and B cells in the surrounding tissues.^35^ In contrast, 10‐month‐old male and female 5xFAD mice also exhibit decreased counts of both CD4^+^ and CD8^+^ T cells in the dural meninges, whereas peripheral organs such as the spleen, lungs, and liver show no significant differences.^21^ These divergent findings highlight potential age‐, sex‐, or methodological dependencies in meningeal immune assessments. Beyond the meninges, the 5xFAD strain also exhibits T‐cell infiltration into the brain parenchyma as early as 6 months of age, with further accumulation at 9^21^ and 12 months,^114^ indicating progressive CNS immune recruitment over time.

Other EOAD models also show CNS T‐cell accumulation. APPswe/PS1^ΔE9^ mice show increased hippocampal T cells by 12 to 13 months of age.^22^, ^115^ In contrast, APP^NL‐G‐F^ mice exhibit an earlier onset of T‐cell accumulation, starting at 3 months,^21^ indicating potentially model‐specific immune kinetics linked to differences in transgene expression, Aβ burden, or antigen presentation.

Unlike the predominantly infiltration‐driven patterns observed in other models, 3xTg‐AD mice exhibit peripheral and central signatures of adaptive immune activation, with minimal early CNS infiltration. These include elevated serum and cortical IgG, increased plasma cells in the spleen and bone marrow, and a Th17‐skewed T‐cell profile in blood and lymphoid tissues.^116^ The absence of brain parenchymal T cells at 3 to 4 months^117^ suggests delayed or restricted CNS immune recruitment in this model.

Thus, adaptive immune engagement is common to EOAD mouse models; however, the timing, anatomical distribution, and qualitative nature of these responses are tightly coupled to strain‐specific mutation profiles and disease kinetics, indicating that immune phenotypes cannot be interpreted independently of pathological context.

### 4.3 Functional implication of depleting adaptive immune cells in EOAD mouse models

If adaptive immunity actively shapes EOAD pathology, then manipulating its function is expected to yield predictable outcomes. Yet, experimental literature reveals that this is not always the case. Various methodological approaches, including genetic manipulation and antibody‐mediated depletion of T and/or B cells, were used to evaluate the impact of adaptive immune cells on Aβ pathology and cognitive function (Table 3, Figure 3). While some studies report exacerbation of pathology following T‐ and B‐cell depletion,^18^, ^19^, ^20^, ^21^ others demonstrate potential neuroprotective effects.^15^, ^16^, ^17^ This conflicting response suggests that adaptive immune cells are neither uniformly protective nor uniformly pathogenic. Instead, their role is likely influenced by various factors, including mutation profile, age and disease stage, and tissue compartment.

**TABLE 3:** Age‐ and subset‐specific effects of adaptive immune manipulations across EOAD mouse models.

Strain	Immune cell types studied	Intervention	Sex	Age, months	Result	Effect of the immune cell type	Reference
5xFAD	T, B, and NK	Genetic (*RAG2*/*IL2* deficiency)	M and F	6	Major increase in Aβ pathology	Protective	^20^
	T, B, and NK	Genetic (*RAG2*/*IL2* deficiency)	M and F	10	Slight increase in Aβ pathology	Protective	^18^
	CD8+	Antibody depletion (anti‐CD8 antibody)	F	8 to 12	Restored behavioral performance to WT level and decreased Aβ pathology in treated 5xFAD mice	Pathogenic	^17^
	B	Genetic (*µMT* ^−/−^ deficiency) and antibody depletion (anti‐CD20 antibody)	Not stated	3	Slightly reduced cognitive performance; increased Aβ pathology	Protective	^19^
	B	Antibody depletion (anti‐CD20 antibody)	F	9	Reduced Aβ pathology	Pathogenic	^15^
	Tregs	Genetic (*Foxp3*‐diphtheria toxin [DTx] receptor [DTR^+^] mice)	M and F	5–6	Reduced Aβ pathology and improved cognitive performance	Pathogenic	^118^
	Tregs	Treatment with p300i	M and F	11	Reduced Aβ pathology	Pathogenic	^118^
	TCRα	Genetic (*TCRα* ^−/−^ deficiency)	F	4 and 10	Reduced cognitive performance at 4 months, increased Aβ pathology at 10 months	Protective	^21^
	MHC I (impaired antigen presentation mainly to CD8^+^)	Genetic (*B2m* deficiency)	M and F	4 and 10	Reduced cognitive performance at 4 months, increased Aβ pathology at 10 months	Protective	^21^
	Brain‐specific CD8^+^ T	Genetic (*CXCR6* deficiency)	M and F	4	Increased Aβ pathology in female 5xFAD CXCR6 deficiency	Protective	^21^
	MHC‐II	Genetic (*MHC‐II* deficiency)	F	1, 3, and 6	Increased Aβ pathology in 5xFAD MHC II^−/−^	Protective	^119^
	MR1/MAIT cell axis	Genetic (*MR1* deficiency)	M and F	2, 4, 6, and 8	Reduced Aβ pathology in 5xFAD MR1^−/−^	Pathogenic	^120^
	CD8^+^ T	Antibody depletion (anti‐CD8a antibody)	M	7.5	Improved cognitive behavior in treated 5xFAD	Pathogenic	^121^
APP/PS1^ΔE9^	B	Genetic (*JHT* deficiency)	F	7	Improved cognitive behavior and reduced Aβ pathology in APP/PS1	Pathogenic	^15^
	CD8^+^	Antibody depletion (anti‐CD8a antibody)	M and F	12 to 13	No effect on Aβ pathology or behavior	No effect	^22^
	T and B	Genetic (*RAG2* deficiency)	M and F	3 and 8	Reduced cognitive performance in RAG2‐deficient mice regardless of APP/PS1 genotype at 8 months; increased Aβ pathology at 3 months and reduced at 8 months in RAG2‐deficient APP/PS1 mice	Protective in 3‐month‐old mice, pathogenic in 8‐month‐old mice	^23^
APP/PS1^L166P^	Treg	Antibody depletion (anti‐CD25 antibody)	Not stated	4 for brain analysis, 7 for behavior assessment	Reduced cognitive performance in Treg‐depleted APP/PS1 mice No effect on Aβ pathology, a decrease in plaque‐associated microglia in Treg‐depleted APP/PS1 mice	No effect in 4‐month‐old mice, protective in 7‐month‐old mice	^122^
	Gut microbiome	Antibiotic treatment	F	5	Reduced Aβ pathology in APP/PS1 mice	Pathogenic	^123^
	IL‐17a	Genetic (*Il17a* deficiency)	F	5	In APP/PS1 Il17a‐deficient mice, antibiotic treatment did not affect Aβ pathology	No effect	^123^
APP^NL‐G‐F^	T and B	Genetic (*RAG2* deficiency) LPS injections to stimulate peripheral inflammation	Mix	5 to 6	Improved cognitive behavior and reduced Aβ pathology	Pathogenic	^16^
	T and B cells	Genetic (*RAG2^fl^* deficiency)	M and F	1 to 4 and 3 to 7	Same effect of microglia depletion in RAG‐2^fl^ APP mice as in WT APP mice; lacking direct statistical analysis of peripheral adaptive immunity factor	Not analysed	^124^
	T and B cells	Genetic (*RAG1*‐deficiency)	M	12	Myelin abnormalities found in APP^NL‐G‐F^ mice were reduced in APP^NL‐G‐F^ RAG1^−/−^ mice	Pathogenic	^121^
3×Tg‐AD	B cells	Genetic (*JHT* deficiency)	F	15	Improved cognitive behavior and reduced Aβ pathology	Pathogenic	^15^
	B cells	Antibody treatment (anti‐CD20 antibody)	F	17	Reduced Aβ pathology	^Pathogenic^	^15^

Abbreviations: ↓ decrease; ↑ increase; CXCR6, C‐X‐C motif chemokine receptor 6; DTx, diphtheria toxin; DTR, diphtheria toxin receptor; Foxp3, forkhead box P3; IL‐17a, interleukin 17a; IL‐2, interleukin 2; LPS, lipopolysaccharide; MHC‐I, major histocompatibility complex class I; MHC‐II, major histocompatibility complex class II; M & F male and female; MR1, MHC class I‐related protein 1; MAIT mucosal‐associated invariant T cells; NK, natural killer; RAG1/2, recombination activating gene 1/2; Treg, regulatory T cell; TCRα, T‐cell receptor α; WT, wild type.

**FIGURE 3:**
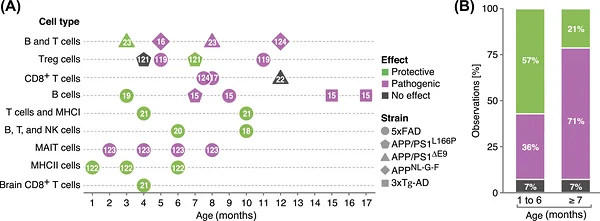
Age and immune cell subset shape adaptive immune effects in AD mouse models. Summary of published observations examining adaptive immune cell populations across AD mouse models, stratified by age, strain, immune cell type, and inferred functional effect of the immune cell population. (A) Observations mapped across age in months for B and T cells, Treg cells, CD8^+^ T cells, B‐cells, T cells and MHCI, B, T, and NK cells, MAIT cells, MHCII cells, and brain CD8^+^ T cells. Symbol shape indicates mouse strain, and color indicates whether the reported effect was protective, pathogenic, or no effect was observed. (B) Summary of proportional distribution of effects in younger mice aged 1 to 6 months versus older mice aged 7 months or more, showing a shift from predominantly protective adaptive immune effects at younger ages toward more pathogenic effects with advancing age.

Further complicating interpretation, sex‐specific effects remain insufficiently resolved. Experimental designs vary considerably, as some studies employ mixed‐sex cohorts,^16^, ^18^, ^20^, ^23^, ^118^, ^124^ other studies restrict analysis to one sex,^15^, ^17^, ^123^ and still others do not report sex at all (Table 3).^19^, ^122^ The absence of direct statistical comparisons between male and female responses to immune modulation further limits the interpretation of sex‐specific effects of adaptive immune targeting. While mutation context and experimental design contribute to variability, the age at which immune manipulation is performed may represent a dominant but underappreciated determinant of outcome.

## 5 ADAPTIVE IMMUNE EFFECTS SHOW AN AGE‐ASSOCIATED SHIFT IN EOAD MODELS

The inconsistencies observed across depletion studies reveal a patterned shift in immune function during disease progression, suggesting that age is a unifying variable. A broad set of studies has examined how depleting distinct adaptive immune cell subsets influences AD pathology in EOAD mouse models. These investigations span multiple mouse strains introduced in Section 4, including 5xFAD, APPswe/PS1^ΔE9^, APP/PS1^L166P^, APP^NL‐G‐F^, and 3xTg‐AD, and use both genetic and antibody‐mediated approaches to target B cells, T cells, CD8^+^ T cells, regulatory T cells (Tregs), NK cells, and related pathways.

To examine whether age was associated with differences in adaptive immune function across EOAD models, we performed a targeted literature search using PubMed and Web of Science, combining terms related to AD mouse models and adaptive immune populations, including “Alzheimer's disease,” “mouse,” “5xFAD,” “APP/PS1,” “APPNL‐G‐F,” “3xTg‐AD,” “adaptive immunity,” “T cell,” “CD4,” “CD8,” “regulatory T cell,” “Treg,” “B cell,” “lymphocyte,” “MHC,” “antigen presentation,” “RAG1,” and “RAG2.” Each distinct age‐specific experimental condition was treated as one observation. Where the same study evaluated closely related immune‐deficiency conditions at the same age and reported concordant outcomes, these conditions were grouped as a single age‐specific observation. Thus, studies examining the same manipulation at different ages could contribute more than one observation. Observations were classified according to the inferred functional effect of the immune cell population, rather than the direction of the experimental intervention. Worsening following depletion or genetic deficiency was classified as evidence of a protective immune function, whereas improvement following depletion or deficiency was classified as evidence of a pathogenic immune function. Analysis of 16 independent studies comprising 28 experimental observations reveals a clear age‐dependent shift in the functional roles of adaptive immune components in AD models (Figure 3A). Among 14 observations reported in mice aged 1 to 6 months, eight (≈57%) describe the protective effects of adaptive immune cells,^19^, ^20^, ^21^, ^23^, ^119^ while five (≈36%) indicate pathogenic roles^16^, ^118^, ^120^ and one (≈7%) shows no effect.^122^ In contrast, among 14 observations from mice aged 7 months or older, 10 (≈71%) are pathogenic,^15^, ^17^, ^23^, ^118^, ^120^, ^121^ three (≈21%) are protective,^18^, ^21^, ^122^ and one (≈7%) shows no effect^22^ (Figure 3B). This analysis is a descriptive synthesis rather than a formal meta‐analysis. Given the substantial heterogeneity across studies in mouse models, immune manipulations, endpoints, and experimental design, individual observations were not weighted by sample size or study quality. Percentages were rounded to the nearest whole number and are presented as descriptive trends rather than pooled estimates of effect.

An important limitation of this comparison is that the included studies differ substantially in the timing and nature of immune manipulation. In particular, knockout mouse models such as RAG2^−/−^ and TCRα^−/−^ mice lack the corresponding lymphocyte populations throughout development. Consequently, phenotypes measured at a given age may reflect developmental compensation or lifelong immune deficiency in addition to the effect of adaptive immunity at that disease stage. Definitive testing of this hypothesis will require temporally controlled immune‐cell depletion or expansion within the same AD model at matched disease stages.

Specifically, at 6 months, a combined *RAG2*/*IL2* deficiency in 5xFAD mice worsened Aβ deposition,^20^ and early *TCRα* or MHC‐I deficiency led to cognitive decline.^21^ In older animals, in contrast, certain interventions targeting defined subsets frequently become protective. Specifically, B‐ or CD8^+^ T‐cell depletion in 8‐ to 12‐month‐old 5xFAD mice improved cognitive performance and reduced Aβ burden.^15^, ^17^ Similarly, *RAG2^−/−^* APP/PS1 mice exhibited increased Aβ pathology at 3 months but reduced pathology by 8 months, directly illustrating a temporal inversion in immune function.^23^

Some interventions, such as mucosal‐associated invariant T (MAIT) cell depletion^120^ or gut microbiome perturbation,^123^ showed protective effects across multiple age groups, suggesting the existence of parallel or co‐interacting mechanisms beyond conventional adaptive immunity. Collectively, these findings demonstrate that adaptive immune activity in EOAD is not static but undergoes stage‐dependent reprogramming.

The contrasting findings from studies of Treg depletion^118^, ^122^ further illustrate that age alone may not fully account for the functional effects of adaptive immune populations. In 5xFAD mice, transient conditional depletion of Tregs at 5 to 6 months reduced Aβ pathology and improved cognitive performance, indicating a pathogenic Treg contribution in this context.^118^ In contrast, anti‐CD25‐mediated Treg depletion in APP/PS1 mice impaired cognitive performance without reducing Aβ pathology, supporting a protective Treg function.^122^ Because these studies differed in AD model, method, and timing of Treg manipulation and the endpoints assessed, it is difficult to determine which factor, or interaction between factors, accounts for the divergent outcomes. Thus, although age emerges as the clearest overall organizing variable across studies, its effects likely interact with model‐ and intervention‐specific factors in shaping adaptive immune function.

Importantly, this analysis is restricted to predominantly Aβ‐driven EOAD models and does not establish that the same age‐associated shift occurs in tauopathy; whether adaptive immune function undergoes a comparable temporal transition in tau‐driven mouse models remains an open question that requires direct investigation.

## 6 ADDITIONAL BIOLOGICAL MODIFIERS: SEX AND GENETIC CONTEXT

Although age and disease stage provide the clearest framework for interpreting adaptive immunity in AD, they do not fully explain immune heterogeneity across patients and models. Sex and genetic context further shape immune composition, signaling thresholds, and inflammatory tone, requiring biologically stratified interpretation of adaptive immune responses.

### 6.1 Sex differences in adaptive immunity in AD

Globally, of the estimated 32.3 million individuals diagnosed with AD, 20.9 million are women and 11.4 million are men, excluding those in prodromal or preclinical stages.^125^ This pronounced sex disparity suggests that biological modifiers beyond age alone contribute to disease susceptibility. Sex‐related differences in adaptive immunity are a compelling mechanistic candidate underlying this imbalance. Compared with men, women generally mount stronger adaptive immune responses, characterized by higher CD4^+^ T‐cell counts and more robust B‐cell‐mediated antibody production. These differences are driven in part by inflammation‐related X chromosome‐linked genes that escape X‐inactivation, thereby amplifying immune signaling in females.^126^ Such baseline immune dimorphism creates a distinct immunological environment in women that may shape the initiation and progression of AD‐relevant neuroinflammatory processes.

The menopausal transition represents a major inflection point in this immune landscape. Because B cells and CD4^+^ T cells express estrogen receptors, adaptive lymphocytes are directly responsive to circulating estrogen levels.^126^, ^127^, ^128^ Declining estrogen can therefore disrupt estrogen receptor‐mediated immune regulation, promoting a more pro‐inflammatory state marked by altered T‐cell ratios, reduced B‐cell numbers, and increased systemic inflammatory signaling.^129^, ^130^ Such remodeling is associated with increased Aβ accumulation and greater vulnerability to AD progression.^131^, ^132^, ^133^, ^134^

Menopausal hormone therapy (MHT) may modify these trajectories,^135^ but its immunological effects are not uniform^136^, ^137^, ^138^, ^139^, ^140^ (Table S1). Estrogen signaling regulates thymic T‐cell development, T‐cell polarization, activation thresholds, B‐cell selection, and tolerance checkpoints,^136^, ^141^, ^142^, ^143^ while other hormones such as progesterone and progestogens further shape adaptive immune responses and T‐cell–microglia interactions.^144^, ^145^ Consistent with this mechanistic framework, clinical and experimental studies demonstrate that MHT measurably reprograms T‐ and B‐cell compartments, with effects that differ according to hormone formulation and route of administration^135^, ^146^, ^147^, ^148^ (Table S1).

These context‐dependent immune effects may help explain divergent associations between MHT and AD risk. While large randomized trials in older postmenopausal women have reported an increased incidence of dementia,^149^ observational studies of MHT initiated in midlife generally report either neutral or protective associations.^150^ Recent integrative reviews further emphasize that the effect of MHT on AD risk depends on initiation near the menopausal transition and APOE ε4 genotype.^150^ These differences are consistent with the immunomodulatory effects of estradiol on T‐cell polarization and B‐cell tolerance described earlier.^141^, ^142^, ^144^, ^151^ Accordingly, regimens that restore estradiol signaling without exacerbating systemic inflammatory pathways or promoting autoreactive B‐cell selection may help preserve a more youthful immune phenotype. Such coordinated immune effects could attenuate chronic low‐grade inflammation and modulate microglia–T‐cell crosstalk, thereby slowing Aβ pathology.^136^ In contrast, late initiation in an already immunosenescent and pro‐inflammatory immune environment may reinforce maladaptive humoral and innate activation and thereby fail to confer, or even worsen, AD risk.^152^ Taken together, these observations support the view that MHT effects on AD risk are determined by the interaction between therapy timing and the underlying immune state rather than by a uniform treatment effect.

### 6.2 Adaptive immunity in DS‐associated AD (DSAD)

While sex differences illustrate how physiological factors shape adaptive immune responses with age, genetic conditions associated with EOAD pathology provide an additional framework for examining immune dysregulation in AD. DS is the most prominent and prevalent example, as individuals with DS develop early amyloid pathology and exhibit chronic immune activation, offering a powerful framework for examining how adaptive immunity contributes to AD pathogenesis in this syndrome. Genetically defined EOAD contexts help distinguish adaptive immune responses driven primarily by amyloid pathology from those amplified by broader genetic perturbation of immunity.

#### 6.2.1 Genetic interferonopathy drives neuroimmune vulnerability in DS

Individuals with DS exhibit a genetically determined predisposition to AD driven primarily by triplication of human chromosome 21 (Hsa21), which results in ∼1.3‐fold increased APP expression and profound immune dysregulation.^153^ Accordingly, Aβ deposition and tau pathology typically emerge in the fourth decade of life, followed by progression to nearly universal dementia by the sixth or seventh decade. Under the revised Alzheimer's Association criteria, individuals with genetically determined AD, including DSAD, are classified as Stage 0 while biomarker‐negative and clinically asymptomatic, reflecting the presence of the underlying disease‐associated genotype from birth.^154^ Beyond APP, the triplicated genome includes additional AD‐related genes such as *dual‐specificity tyrosine‐phosphorylation‐regulated kinase 1A (DYRK1A)*, whose overexpression disrupts endosomal trafficking, promotes aberrant APP processing, enhances tau phosphorylation, and contributes to synaptic dysfunction.^155^

DS provides a uniquely informative context for understanding how genetically driven amyloid burden intersects with immune dysregulation to accelerate AD pathophysiology.^156^, ^157^ A central and parallel feature of trisomy 21 is constitutive type I IFN (IFN‐I) hypersensitivity. Triplication of IFN‐α and β receptor subunits 1 and 2 (*IFNAR1* and *IFNAR2*) increases receptor expression by ∼50%, causing physiological levels of IFN‐α and β to trigger exaggerated Janus kinase‐signal transducer and activator of transcription (JAK‐STAT) signaling and chronic induction of IFN‐stimulated genes. This response is further amplified by the triplication of Hsa21‐encoded antiviral IFN‐stimulated genes, including MX dynamin‐like GTPase 1 and 2 (*MX1* and *MX2*) and IFN‐induced transmembrane protein 1 and 3 (*IFITM1* and *IFITM3*), which enhance downstream transcriptional activation. These gene–dosage effects create a lifelong interferonopathy detectable from infancy and characterized by heightened basal inflammation and premature immune aging.^158^

This chronic IFN‐I signaling, in turn, reshapes the adaptive immune system in ways that are implicated in AD pathogenesis. IFN‐I accelerates thymic involution, reduces naïve T‐cell output, and drives early expansion of effector and TEMRA T cells, hallmarks of advanced immunological aging. These highly differentiated T‐cell populations exhibit increased cytotoxicity and elevated IFN‐γ secretion, thereby lowering the activation threshold of microglia and promoting pro‐inflammatory microglial states. Similarly, lifelong exposure to IFN‐I disrupts B‐cell maturation and class switching, reduces naïve B‐cell pools, and promotes exhausted or autoreactive B‐cell phenotypes, producing a humoral compartment that is hyperactivated yet functionally inefficient, a pattern that mirrors B‐cell dysregulation observed in sporadic AD but emerges decades earlier in DSAD.^153^

#### 6.2.2 Neuroimmune amplification of AD pathology in DS

Within the CNS, chronic IFN‐I signaling drives microglia toward a maladaptive activation state. Although acute IFN‐I exposure can transiently enhance Aβ clearance,^159^ sustained signaling progressively disrupts lysosomal pathways, reduces phagocytic efficiency, and increases pro‐inflammatory cytokine release, thereby promoting both Aβ accumulation and tau phosphorylation. As a result, IFN‐I hypersensitivity establishes a feedforward loop in which peripheral adaptive immune aging exacerbates microglial dysfunction,^160^ while microglial stress further promotes the recruitment and activation of cytotoxic T‐cells at the brain borders.^161^ These alterations produce systemic inflammation, impaired immune regulation, and accelerated immunosenescence that closely mirror and markedly intensify the immune perturbations observed in sporadic AD, thereby accelerating AD neurodegeneration in DS. These converging neuropathological and immunological features position DS as a genetically defined framework for investigating early AD mechanisms.^156^, ^162^

#### 6.2.3 APP duplication as a minimal amyloid‐driven immune context

In contrast to DS, duplication of the *APP* gene (located on chromosome Hsa21) represents a genetically defined form of EOAD characterized by increased *APP* dosage in the absence of triplication of immune‐related genes.^163^, ^164^ Individuals with *APP* duplication develop early and robust Aβ pathology, similar to that observed in DS, but without the constitutive IFN signaling and systemic immune dysregulation associated with trisomy 21.^165^ Accordingly, *APP* duplication may reflect a “minimal” model of amyloid‐induced immune engagement, whereas DS represents a “maximal” neuroimmune context in which amyloid pathology and intrinsic immune dysregulation converge.^163^ Comparing these conditions may therefore help clarify the extent to which adaptive immune responses in AD are triggered by Aβ itself or shaped by underlying immune‐genetic background.

### 6.3 Therapeutic implications and clinical translation

The oligomerization of Aβ monomers and their subsequent aggregation into neuritic plaques occur silently during a decades‐long presymptomatic phase, followed by inflammation and tau pathology that drive neurodegeneration and clinical symptoms. This temporal trajectory suggests that anti‐Aβ therapies are likely to be the most effective when initiated early in the disease course, before irreversible neuronal damage occurs.^52^, ^166^ As immune‐based therapies have progressed from conceptual strategies to clinical implementation, it has become evident that the manner in which adaptive immunity is engaged critically determines both therapeutic efficacy and safety. Interestingly, population‐level evidence suggests that immune interventions not specifically directed against AD pathology may also influence dementia risk. In a natural experiment exploiting age‐based eligibility for herpes zoster vaccination, receipt of a vaccine was associated with a significant reduction in the probability of a new dementia diagnosis over 7 years.^167^

Early attempts to harness adaptive immunity demonstrated that antibody‐mediated clearance of Aβ is feasible. However, these studies also exposed the risks associated with the unintended activation of pro‐inflammatory T‐cell pathways.^14^ These early safety signals have guided the development of next‐generation immunotherapies that aim to elicit robust and durable humoral responses with minimal cellular autoreactivity. In parallel, studies isolating memory B cells reactive to Aβ from patients with AD provide functional evidence that an adaptive immune, B‐cell‐dependent response against amyloid pathology can be effectively generated in humans. These observations complement reports of naturally occurring anti‐Aβ antibodies and further strengthen the biological rationale for antibody‐based therapeutic strategies.^166^, ^168^ Within this evolving therapeutic landscape, two principal immunological strategies have emerged: active vaccination, which seeks to induce sustained cellular and endogenous polyclonal humoral anti‐Aβ and/or anti‐tau responses, and passive immunization, which delivers highly specific monoclonal antibodies to accelerate amyloid removal with controlled target engagement. Understanding how these complementary approaches mobilize adaptive immunity provides essential context for evaluating their clinical outcomes and for interpreting the variability in efficacy and safety observed across disease stages and patient populations.

### 6.4 Active vaccination: humoral engagement versus T‐cell liability

One of the earliest landmark efforts in AD immunotherapy was the AN1792 trial, which evaluated a full‐length Aβ_42_ active first‐generation vaccine formulated with the QS‐21 adjuvant, developed by Elan Pharmaceuticals. Although AN1792 generated functional anti‐Aβ antibodies that were associated with plaque clearance in responders, the phase 2a trial with this vaccine was halted prematurely due to meningoencephalitis in ∼6% of participants^14^ (Table 4). Subsequent analyses demonstrated that these adverse events were driven by a pro‐inflammatory, Th1‐skewed T‐cell response, particularly in participants who received a formulation containing polysorbate 80.^13^ This experience exemplified the need for vaccine designs that elicit robust B‐cell‐mediated humoral immunity while avoiding autoreactive T‐cell activation. Although AN1792 generated functional anti‐Aβ antibodies and evidence of amyloid clearance, long‐term neuropathological follow‐up demonstrated that substantial, and in some cases nearly complete, plaque removal did not prevent progression to severe dementia or ongoing neurodegeneration.^169^ This dissociation between amyloid removal and clinical progression supports the possibility that Aβ‐directed intervention may have limited efficacy once downstream neurodegenerative processes are established.

**TABLE 4:** Comparison of active Aβ vaccines in AD clinical development.

Feature	AN1792	UB‐311	ACI‐24.060
Type	Active immunotherapy (full‐length Aβ_42_ peptide + QS‐21 adjuvant)	Active immunotherapy (synthetic peptide vaccine with Th‐cell epitopes)	Active immunotherapy (liposomal vaccine presenting Aβ1‐15 peptide in β‐sheet conformation)
Target	Aβ (Aβ_42_)	Aβ (specific epitopes, avoids autoreactivity)	Aβ oligomers and pyroglutamate‐modified species
Mechanism	Induced Th‐1‐skewed broad antibody response against Aβ	Induces Th2‐skewed immune response, avoids pro‐inflammatory T‐cell response	Designed to generate antibodies against toxic Aβ oligomers, including pyroglutamate forms
Clinical stage	Phase 2a (stopped early in 2002 due to meningoencephalitis in ∼6% of patients)	Phase 2 (in mild AD; earlier trials showed safety and tolerability)	Phase 1/2 in DS and AD (updated liposomal formulation, 2023‐ongoing trials)
Efficacy	*Post mortem* studies showed reduced plaque burden, but no clear cognitive benefit	Showed good immunogenicity, safe in the elderly; some slowing of cognitive decline was reported in phase 2a	Preclinical and early human data suggest strong antibody response; clinical efficacy still under investigation
Safety	Major safety issue: T‐cell‐mediated meningoencephalitis → trial halted	Good safety profile so far; no serious autoimmune reactions	So far, safe and well‐tolerated in early trials
Status	Discontinued	Ongoing (Phase I)	Ongoing (Phase 1/2)
References	^13^, ^14^, ^169^	^12^	^11^

Abbreviations: DS, Down syndrome; Th, T helper.

In response, second‐generation active vaccines have been engineered and are currently in clinical development. UB‐311, a synthetic peptide vaccine developed by United Biomedical, is engineered to induce a predominantly B‐cell‐driven, Th2‐skewed immune response. In a phase 2a trial in mild AD, it elicited antibody responses in >90% of participants and demonstrated a favorable safety profile^12^ (Table 4). ACI‐24.060, a liposome‐based vaccine developed by AC Immune that presents the Aβ_15_ epitope in a β‐sheet conformation, is similarly designed to promote humoral immunity while minimizing T‐cell‐mediated inflammation.^11^ Notably, this program includes participants with DS, recognizing their unique vulnerability to AD. Interim data from the ongoing ABATE trial (a phase 1b/2 study evaluating ACI‐24.060 in AD and DS populations) indicate early induction of anti‐Aβ antibodies, although quantitative titers, durability, and clinical impact remain under evaluation (Table 4). In parallel, additional peptide‐based vaccine platforms have sought to refine Aβ targeting and minimize the risk of autoreactive T‐cell activation. CAD106 (Novartis) and ACC‐001 (Janssen) both employed short N‐terminal Aβ epitopes conjugated to carrier proteins and achieved robust anti‐Aβ antibody responses with acceptable tolerability, though phase 2 development was discontinued in the absence of clear clinical benefit. More recently, ABvac40, a vaccine targeting the C‐terminus of Aβ_40_, completed a phase 2 trial in early AD, demonstrating good tolerability, durable antibody titers, and exploratory signs of benefit on cognition and brain atrophy, particularly in relation to vascular amyloid burden.^170^

In addition to Aβ‐directed strategies, tau‐targeting vaccines are emerging as a complementary approach that directly engages adaptive immunity against the protein species most tightly linked to neurodegeneration. AADvac1, an active vaccine directed against a pathological microtubule‐binding region of tau, demonstrated good safety and strong antibody responses in the ADAMANT phase 2 clinical trial, with post hoc analyses suggesting potential benefit in biomarker‐defined subgroups characterized by elevated plasma p‐tau217.^171^ Importantly, the evaluation of the AADvac1 vaccine includes a combination with anti‐Aβ monoclonal antibodies. Similarly, the liposomal vaccine ACI‐35.030 and its conjugated analog JACI‐35.054, designed to present phosphorylated tau (pTau) epitopes in an immunogenic configuration, have elicited strong pTau‐specific humoral responses with acceptable tolerability in early‐phase trials.^172^

Collectively, these candidates demonstrate the field's progression from full‐length Aβ vaccination toward epitope‐focused, B‐cell‐driven approaches with increased efforts toward combined targeting of tau pathology. This progression reflects a broader effort to maximize immunogenicity while minimizing T‐cell‐mediated neuroinflammation and to extend adaptive immune targeting beyond amyloid to downstream drivers of neurodegeneration.

### 6.5 Passive immunization: early‐stage benefit and amyloid‐related imaging abnormality (ARIA)‐defined limits

Passive Aβ‐targeting immunotherapies have reshaped the therapeutic landscape of AD. Aducanumab (the EMERGE/ENGAGE trials),^173^ lecanemab (the CLARITY‐AD trial),^174^ and donanemab (the TRAILBLAZER‐ALZ 2 trial)^175^ have each demonstrated robust Aβ‐plaque clearance and modest, but statistically significant, slowing of cognitive and functional decline, with the strongest effects observed in patients at earlier disease stages.

Lecanemab received full US Food and Drug Administration (FDA) approval in 2023 after demonstrating clinical benefit in early symptomatic AD. Following 18 months of treatment, cognitive decline was reduced by 27%, as measured by the Clinical Dementia Rating–Sum of Boxes, with consistent benefits across additional cognitive and functional scales.^166^ Donanemab received full FDA approval in July 2024 for patients with mild cognitive impairment or mild AD with confirmed Aβ pathology, based on phase 3 data showing slowed cognitive and functional decline, particularly among individuals with intermediate tau pathology.^166^ Both lecanemab and donanemab are associated with significant adverse effects, most notably ARIA, and are therefore recommended primarily for carefully selected early‐stage patients following genetic and biomarker screening.^166^ ARIA are thought to arise partly from antibody‐mediated mobilization of Aβ in the presence of vascular amyloid/CAA, which can promote vascular inflammation, BBB disruption, edema (ARIA‐E), and hemorrhagic lesions (ARIA‐H).^176^ Aducanumab, previously granted accelerated approval based primarily on its Aβ‐lowering effect, was fully discontinued in 2024, with Biogen halting both development and commercialization after years of controversy and limited clinical uptake.^166^ Lecanemab and donanemab now represent the standard Aβ immunotherapies with full FDA approval, whereas aducanumab is no longer available.^177^, ^178^, ^179^ Newer delivery strategies aim to improve brain exposure while limiting vascular toxicity. Trontinemab, an anti‐Aβ antibody fused to a transferrin receptor 1‐targeting Brainshuttle, has shown rapid amyloid plaque clearance with a low observed incidence of ARIA in early‐phase studies, although larger trials are required to establish its long‐term safety and clinical efficacy.^180^

Beyond these agents, a new generation of high‐affinity anti‐Aβ antibodies is advancing through late‐stage development. Among these, remternetug is entering phase 3 trials and is designed to achieve more rapid plaque clearance, along with more convenient subcutaneous administration. However, it remains uncertain whether these pharmacodynamic advances will translate into greater clinical benefit or meaningfully alter ARIA risk.^181^

### 6.6 Antibody‐ and fragment crystallizable (Fc)‐mediated mechanisms beyond canonical anti‐Aβ monoclonal antibodies

Intravenous Ig (IVIG) is a therapeutic preparation consisting of pooled IgG antibodies collected from the plasma of thousands of healthy blood donors,^182^ encompassing a broad repertoire that includes naturally occurring antibodies against Aβ and other self‐antigens. In AD, IVIG has been explored for its potential to neutralize toxic Aβ species, modulate microglial activation, and restore immune homeostasis. The majority of Aβ‐binding natural antibodies within IVIG are conformation‐selective, preferentially recognizing oligomeric and fibrillar forms of Aβ (see review by Relkin^183^). A phase 3 clinical trial of IVIG administration to individuals with mild to moderate LOAD did not demonstrate a cognitive benefit.^184^ In contrast, preclinical studies further indicate that IVIG effects are variable and depend on treatment timing and disease context. For example, in 19‐month‐old APP/PS1 mice, peripheral IVIG administration increased BBB permeability and antibody infiltration into the brain parenchyma, with colocalization to Aβ plaques, and enhanced microglial clearance of Aβ ex vivo.^185^ In contrast, prolonged IVIG treatment initiated at 4 months of age did not reduce Aβ burden despite increased infiltration of CD45^+^ immune cells.^186^ In young 3xTg‐AD mice, early IVIG treatment corrected the elevated CD4/CD8 T‐cell ratio and reduced the oligomeric Aβ levels.^187^ In the same model, IVIG treatment was also associated with reduced tau pathology.^188^ Intracranial IVIG reduced brain Aβ levels and was associated with decreased inflammatory signaling and increased neurogenesis across AD mouse models.^189^, ^190^

While Igs have been implicated in modulating pathology in EOAD mouse models, direct causal evidence remains limited, as no studies have investigated AD‐related outcomes in Ig‐deficient EOAD mouse models. Consistent with the lack of clinical efficacy in established LOAD, preclinical findings suggest complex, model‐ and timing‐dependent effects that warrant further investigation into its mechanisms and potential efficacy at earlier disease stages.

Given that the biological activity of IVIG and other Ig‐based interventions is largely mediated through Fc‐Fcγ receptor (FcγR) interactions,^191^ understanding FcγR expression and signaling within the CNS is essential for interpreting their effects in AD. Importantly, findings from human‐derived IVIG studies in mouse models should be interpreted with caution due to species‐specific differences in Ig‐FcγR binding affinity and downstream signaling.^192^ Further, the ability of IVIG to engage activating and inhibitory FcγRs determines whether downstream responses are anti‐inflammatory and neuroprotective or, conversely, pro‐inflammatory and neurotoxic.^193^ Thus, while IVIG studies highlight the therapeutic potential of antibody‐mediated mechanisms, they also underscore the need to consider the local FcγR landscape within the brain as a key modulator of Ig function and outcome in AD.

Several FcγR‐related mechanisms have been proposed in AD pathogenesis, reflecting the diverse ways in which Ig‐FcγR interactions can influence neuronal survival and neuroinflammation (see review by Fuller et al.^193^). These mechanisms suggest that Ig‐FcγR signaling can promote either neurodegeneration or immune‐mediated clearance depending on context. Importantly, combined *APOE* and FcγR developmental deficiency in mice confers protection against neuropathological and cognitive alterations compared with *APOE*‐deficient mice alone.^194^ These reports underscore the need for more systematic investigation of Ig involvement in AD etiology. Accordingly, the use of Ig‐deficient mouse models may help clarify the precise contribution of Ig to AD pathogenesis.

### 6.7 DS as a stratified translational setting

Historically, individuals with DS have been systematically excluded from pivotal AD trials, such as those evaluating anti‐Aβ monoclonal antibodies, including lecanemab and donanemab, largely because of eligibility criteria that indirectly resulted in disqualification from the trials. Specifically, many studies excluded participants with intellectual disabilities, imposed age ranges that were misaligned with the earlier onset of AD in individuals with DS, and relied on cognitive outcome measures that have not been validated for this population (see review by Barroeta et al.^156^). In recent years, however, the field has begun to address this gap by developing disease‐modifying and symptomatic clinical trials specifically tailored to the distinctive biology of DSAD.

The Trial‐Ready Cohort for DS (TRC‐DS) (NCT04165109), initiated under the Alzheimer's Clinical Trials Consortium (ACTC)‐DS network, has established the biomarker infrastructure and recruitment platform that enables intervention studies in adults with DS aged 25 to 55. Building on this preparatory framework, several targeted interventional programs are now under way. The ABATE trial (NCT05462106) began enrollment in 2024 to evaluate ACI‐24.060, an active anti‐Aβ vaccine in asymptomatic or prodromal individuals with DS (ages 35 to 50), and reported favorable interim safety data by late 2024. The HERO study (NCT06673069), launched in 2024, is a phase 1b single‐ascending‐dose trial of ION269, an antisense oligonucleotide that suppresses APP‐derived Aβ production in amyloid‐positive adults with DS (35 to 55 years). The forthcoming ALADDIN trial (NCT06911944), which opened recruitment in 2025, will evaluate donanemab in individuals with DS (35 to 50 years), with particular emphasis on safety and on monitoring ARIA through rigorous magnetic resonance imaging protocols. Complementing these disease‐modifying efforts, parallel strategies are targeting symptomatic and network‐level dysfunction in DSAD. The LESS‐AD study (NCT07234695), coordinated by Hospital de Sant Pau in Spain, aims to evaluate whether levetiracetam, a drug typically used to prevent epilepsy, can delay neurodegeneration and support seizure prevention in individuals with DSAD. It is currently recruiting participants as of 2025. Collectively, these emerging clinical trials represent a pivotal step in the therapeutic development pipeline for DSAD. By integrating biomarker‐driven designs with immune‐targeted interventions within a genetically defined population, these studies offer a powerful translational framework to dissect how adaptive immune alterations influence amyloid processing, neuroinflammation, and disease progression.

### 6.8 Toward stage‐ and context‐specific immunomodulation in AD

The collective evidence reviewed here indicates that adaptive immunity in AD cannot be effectively targeted using uniform therapeutic strategies. Across human studies, genetic analyses, experimental models, and clinical trials, adaptive immune responses are not fixed in function but dynamically evolve across disease progression. Age and disease stage consistently emerge as the most reliable determinants of whether adaptive immune activity is protective or pathogenic. In contrast, sex, genetic background, and baseline immune state further modulate these effects. These observations argue for a shift from generalized immune targeting toward a precision immunomodulation framework in which therapeutic interventions are aligned with the immunological and pathological state of the individual. Immune checkpoint modulation provides a particularly illustrative example of this context dependence. Systemic programmed cell death protein 1 (PD‐1) blockade induced an IFN‐γ‐dependent immune response, promoted recruitment of peripheral monocyte‐derived macrophages to the brain, reduced Aβ pathology, and improved cognitive performance in AD mouse models.^195^ However, these findings were not reproduced in a subsequent study, which observed peripheral immune activation following anti‐PD‐1 treatment but no detectable monocyte‐derived macrophage infiltration into the brain or reduction in cerebral Aβ pathology across several amyloid mouse models.^196^

The first principle of such an approach is *temporal specificity*. Evidence from EOAD mouse models demonstrates that early in the disease course, adaptive immune components often support protective functions, including immune surveillance, modulation of microglial activity, and facilitation of Aβ clearance. In contrast, at later stages, the same immune compartments can become maladaptive, contributing to chronic inflammation, impaired clearance mechanisms, and neurotoxicity. Clinical data are consistent with this pattern, as anti‐Aβ monoclonal antibodies show the greatest efficacy when administered in early symptomatic or prodromal stages, whereas interventions applied later yield limited benefit. These findings suggest that the timing of immune engagement is not merely a practical consideration but a central determinant of therapeutic outcome.

The second principle is *cell type specificity*. Adaptive immunity in AD is not a single axis but a composite of functionally distinct cell populations with divergent roles. Tregs, for example, may restrain excessive inflammation and support homeostasis in early disease, whereas cytotoxic or other pathogenic CD8^+^ T cells and certain B‐cell subsets may contribute to pathology at later stages. This concept has begun to translate into clinical research. Low‐dose IL‐2, which preferentially expands Tregs, was shown to be safe and to increase circulating Treg numbers in a phase 1 study of patients with AD.^197^ More recently, a randomized phase 2a trial demonstrated increased Treg abundance and suppressive function together with changes in inflammatory and AD‐related CSF biomarkers, supporting the feasibility of therapeutically restoring Treg activity in AD.^198^ Similarly, the evolution of vaccine design from full‐length Aβ immunization to epitope‐focused, B‐cell‐driven strategies reflects an increasing recognition that selective engagement of humoral immunity, while minimizing pro‐inflammatory T‐cell activation, is critical for safety and efficacy. However, a detailed, stage‐resolved map linking specific T‐ and B‐cell subsets to protective versus pathogenic functions remains lacking. Defining these relationships represents a major barrier to rational therapeutic design.

The third principle is *host stratification*. Adaptive immune responses in AD are shaped by systemic biological context. Sex‐related immune dimorphism, particularly the immunological consequences of menopause, alters T‐cell polarization, B‐cell function, and inflammatory tone. Genetic factors, including *APOE* genotype and immune‐related risk loci, further influence antigen presentation, lymphocyte activation, and microglial interactions. In parallel, conditions such as DS illustrate how chronic immune activation and IFN signaling can profoundly reshape adaptive immunity and accelerate AD pathology. These factors indicate that patients do not share a uniform immune landscape and that effective immunomodulation will require stratification based on biological variables that influence immune function.

## 7 OPEN QUESTIONS AND FUTURE DIRECTIONS

Despite rapid advances, our understanding of how adaptive immunity shapes AD pathogenesis remains incomplete. Future research must determine when and through which mechanisms adaptive immune responses shift from protective or regulatory roles toward pathogenic activities and define the pathophysiological conditions under which these transitions occur, including the cellular and temporal dynamics of B‐ and T‐cell activity across disease progression.

First, does biological age itself causally alter the function of adaptive immune cells during AD progression? The age‐associated pattern identified across EOAD models suggests that adaptive immune populations may exert protective effects earlier in disease and increasingly pathogenic effects at later stages. However, many existing studies rely on constitutive immune deficiencies, such as RAG‐ or TCR‐deficient mice, in which lymphocyte populations are absent throughout development. Such models cannot distinguish the effects of immune aging from developmental compensation or lifelong immune deficiency. This hypothesis therefore requires direct testing using temporally controlled depletion or expansion of defined immune‐cell populations within the same AD model at matched early and late disease stages. Complementary heterochronic lymphocyte‐transfer experiments, in which lymphocytes from young and aged donors are transferred into age‐matched or age‐mismatched recipients, could distinguish cell‐intrinsic effects of immune aging from those imposed by the aged systemic or CNS environment. Parabiosis approaches could similarly determine the extent to which circulating age‐associated factors reprogram adaptive immune function. Importantly, the present age‐associated analysis is restricted predominantly to Aβ‐driven EOAD models and does not establish that the same temporal shift occurs in tauopathy; comparable age‐resolved experiments in tau‐driven models will therefore be essential.

Second, which antigen‐specific T‐ and B‐cell populations drive protective versus pathogenic responses, and where are these responses initiated? It remains unclear which CD8^+^ T‐cell populations mediate neurotoxicity versus immune surveillance, which B‐cell subsets support protective antibody responses rather than autoreactivity, and whether these functions differ between amyloid‐ and tau‐dominated disease states. Likewise, the antigens driving clonal T‐ and B‐cell expansion remain incompletely defined. Longitudinal paired TCR‐ and BCR‐repertoire sequencing across blood, CSF, meninges, cervical lymph nodes, and brain could determine whether expanded clones are shared across anatomical compartments and how their abundance and phenotype change with disease progression. Combining repertoire analysis with single‐cell transcriptomics, spatial profiling, and unbiased antigen‐discovery approaches will be required to identify the cognate antigens driving these clones and determine whether they preferentially recognize Aβ, tau, microbial antigens, or other CNS‐derived targets. Such studies could also establish whether adaptive immune priming occurs primarily in cervical lymph nodes, meningeal immune niches, or other peripheral compartments and why antigen exposure promotes tolerance in some contexts but sustained inflammatory responses in others.

Third, can stage‐specific adaptive immune states be translated into biomarkers that guide therapeutic intervention? Adaptive immune responses in AD are shaped not only by disease stage but also by sex, genetic background, and baseline immune state. Sex‐linked immune differences, X‐chromosome escapee genes, and risk alleles such as APOE and HLA‐DRB1 may collectively establish distinct immunological set points that influence how adaptive immunity responds to emerging pathology. Genetically defined contexts such as DS may provide an additional framework for dissecting these interactions, particularly by comparing it with APP duplication to distinguish amyloid‐driven immune activation from broader immune‐genetic dysregulation. Longitudinal human studies should therefore integrate peripheral and CSF immune profiling with Aβ and tau biomarkers, age, sex, genotype, and clinical disease stage to identify immune signatures associated with protective versus pathogenic states. Ultimately, immune‐stratified clinical trials will be required to determine whether selecting patients by immunological state, rather than diagnosis alone, improves the efficacy and safety of immune‐directed therapies.

## 8 CONCLUSION

The body of evidence presented herein demonstrates that adaptive immunity is a central and multifaceted contributor to AD pathophysiology. Human studies show that T‐ and B‐cell responses engage AD‐relevant brain compartments, with T cells more closely associated with tau pathology and B cells/antibodies linked primarily to Aβ burden. Complementing these observations, GWASs have identified genetic loci linked to adaptive immune processes, reinforcing the mechanistic relevance of antigen presentation, lymphocyte regulation, and Ig‐mediated pathways. Experimental evidence further supports this framework as findings from EOAD mouse models illustrate that adaptive immune activity is highly context‐ and age‐dependent.

Together, these insights suggest that adaptive immunity is neither uniformly detrimental nor uniformly protective in AD but instead operates along a dynamic spectrum shaped by intrinsic and extrinsic factors. This spectrum is further modulated by host‐specific biological contexts. Additional modifiers, including sex‐related immune dimorphism, genetic risk architecture, and IFN‐driven immune remodeling observed in DS, illustrate how host‐specific factors shape adaptive immune behavior across the AD spectrum. Accordingly, therapeutic strategies aimed at modulating adaptive immunity will require careful tailoring, particularly with respect to the stage of the disease at which such interventions are introduced.

## CONFLICT OF INTEREST STATEMENT

The authors declare no conflicts of interest. Author disclosures are available in the Supporting Information.

## Supporting information




Supporting Information

Supporting Information
