# Supplementary material for: Age‐linked biological context shapes adaptive immunity in Alzheimer's disease
Source: Alzheimers Dement. 2026 Nov 6;22(11):e71978. doi: 10.1002/alz.71978 (PMC13741854; doi:10.1002/alz.71978)
Supplement: Supplementary file 2 [file ALZ-22-e71978-s002.docx]

**Table S1. Formulation- and route-dependent immunomodulatory effects of menopausal hormone therapy (MHT).**

| **Factor** | **Condition** | **Immune Effects** | **References** |
| --- | --- | --- | --- |
| **Route of administration** | Oral MHT (e.g., CEE ± MPA) | ↑ CD80⁺ monocytes; ↑ NK and B-cell numbers; ↑ CRP; ↓ MCP-1; altered monocyte phenotype | [135,146,147] |
|  | Transdermal MHT (17β-E2) | ↑ CD206^+^ monocytes; ↑ CD4⁺ Th cells; partial restoration of CD4⁺/CD8⁺ ratio; less pro-inflammatory profile; minimal effect on CRP; ↓ MCP-1; altered monocyte phenotype | [135,146,147] |
| **Estrogen formulation** | 17β-E2 | Shifts from Th1/Th17 towards Th2 and Tregs; anti-inflammatory adaptive immune profile | [136] |
|  | CEE | ↑ hepatic acute-phase and inflammatory markers (including CRP) | [138,139,146, 147] |
| **Progestogen effects** | Synthetic progestins (e.g., MPA) | Alter IL-6–CRP coupling; associated with IL-6–CRP correlation; may shift inflammatory signaling depending on dose and context | [138,139] |
|  | Micronized progesterone | Does not increase CRP; no additional inflammatory burden when combined with non-oral E2 | [140] |
| **Dose / host factors** | Dose-, age-, BMI-dependent | Variable CRP response; potential pro-inflammatory shift at higher dose | [149] |

Abbreviations: body mass index (BMI); conjugated equine estrogen (CEE); C-reactive protein (CRP); cluster of differentiation 4 (CD4); cluster of differentiation 8 (CD8); cluster of differentiation 80 (CD80); cluster of differentiation 206 (CD206); 17β-estradiol (17β-E2); interleukin-6 (IL-6); medroxyprogesterone acetate (MPA); menopausal hormone therapy (MHT); monocyte chemoattractant protein-1 (MCP-1); natural killer (NK); T helper (Th); regulatory T cells (Tregs). ↑ increase; ↓ decrease.
